# Central role of the sentinel acute pancreatitis event (SAPE) model in understanding recurrent acute pancreatitis (RAP): Implications for precision medicine

**DOI:** 10.3389/fped.2022.941852

**Published:** 2022-08-15

**Authors:** David C. Whitcomb

**Affiliations:** ^1^Cell Biology and Molecular Physiology, and Human Genetics, Division of Gastroenterology, Hepatology and Nutrition (Chief 1999-2016), University of Pittsburgh and UPMC, Pittsburgh, PA, United States; ^2^Ariel Precision Medicine, Pittsburgh, PA, United States

**Keywords:** chronic pancreatitis in children, precision medicine, genetics, recurrent acute pancreatitis, CFTR (cystic fibrosis transmembrane conductance regulator), CFTR-related disorders (CFTR-RD), alcohol, smoking

## Abstract

Traditional approaches to understanding the origins of chronic pancreatitis (CP) and find treatments led to abysmal failure. Thus, no drugs now exists to meet this need. Outdated concepts of the etiopathogenesis of CP have been replaced with new insights and disease models that provide the framework for early detection of the pathogenic pancreatitis process. Application of these principals require a new paradigm in disease definition and management, i.e. personalized / precision medicine. The key is acute pancreatitis (AP) starting with the first (sentinel) acute pancreatitis (AP) event (SAPE). This event sensitizes the pancreas to recurrent acute pancreatitis (RAP) as ongoing stressors drive various inflammatory responses to cause CP. The problem is the complex etiologies of AP and the additional genetic and environmental factors that promote progression to RAP and CP. This paper provides a background on the key conceptual changes that facilitate new approaches and the rationale for using mechanism-specific therapies to prevent RAP and CP.

## Introduction

There have been very few new treatments approved for the prevention of recurrent acute pancreatitis (RAP) or chronic pancreatitis (CP) in humans. The problem is complex and previously unsolvable, as the historical definitions and models of CP were wrong, and the translation from case-control studies, cohort studies and population-based epidemiology studies to the specific therapeutic needs of individual patients at a specific point of time is, frankly, impossible. Indeed, after 100 years of pancreatic research since the suggestion by Chiari that pancreatitis was linked to autodigestion of the pancreas by pancreatic digestive enzymes in 1896 (1) the leading experts finally concluded that CP “remains an enigmatic process of uncertain pathogenesis, unpredictable clinical course, and unclear treatment” (2). This review highlights the fact that the *traditional disease paradigm* required to understand CP was wrong. A more effective approach than the traditional approaches of modern Western Medicine based on the Germ Theory of Disease is the paradigm of Precision Medicine (3, 4).

## Traditional CP definitions, diagnostic criteria and etiologic theory

We begin by highlighting three basic problems with the traditional disease paradigm.

### Problem 1

The first problem was the traditional pathophysiological definition of CP. The famous Marseille-Rome classification conference of 1989 defined CP as “the presence of chronic inflammatory lesions characterized by the destruction of exocrine parenchyma and fibrosis and at least in the later stages, the destruction of endocrine parenchyma.” (5) This is a descriptive-pathologic definition of an advanced inflammatory disease used to distinguish CP from other diseases with similar features of pancreatic inflammation, fibrosis, atrophy, and Type 1 or Type 2 diabetes mellitus. It requires advanced disease features and pathology as a the primary state, but fails to define the essence of the underlying disorder or the unique pathogenic processes.

### Problem 2

The second problem was diagnosis. Since tissue pathology is generally not available for diagnosis, the Pancreatic Society of Great Britain and Ireland developed clinically applicable diagnostic criteria for CP during the 1984 Cambridge Conference (6) using imaging findings of *irreversible pancreatic fibrosis* as the defining feature of CP. These descriptive definitions and corresponding diagnostic criteria are useful for clinical documentation of underlying disease, but (a) require the underlying disease to advance to an irreversible stage before it can be diagnosed, and (b) provides no insights into disease mechanisms or targets for early therapy. However, fibrosis alone is not necessary or sufficient for distinguishing CP form other conditions in the differential diagnosis, especially at earlier stages when treatment may still be effective (7).

### Problem 3

The third problem was etiology. The primary etiology for human CP was believed to be alcoholism, with alcohol typically associated with 80% of cases (8–11). However, a few studies reported that 50% or more of the cases of CP in adults were idiopathic (11–15). The idiopathic group also increased in overall fraction of CP cases as abdominal imaging technology improved, identifying CP cases at earlier stages. But even with alcoholic etiology, <5% of alcoholics develop CP (16) and animals fed alcohol do not get acute pancreatitis (AP) or CP (17, 18) unless pancreatitis is driven by other factors, such as cerulean in alcohol-drinking animals (19). Thus, the primary “etiology” of CP cannot not be the direct cause.

In summary, at least three fundamental clinical concepts surrounding CP were known problems without clear solutions. This resulted in persistence of two major barriers to treating CP: lack of clarity on the mechanistic process leading from a normal-appearing, completely asymptomatic human to end-stage CP and therapeutic targets of the pathogenic mechanism(s) are needed for effective intervention—especially when the disease is in an early stage to stop progression.

## A breakthrough that required a complete paradigm shift in modeling CP

Hereditary pancreatitis (HP) is an autosomal dominant, high-penetrance form of RAP/CP. A study of large families with HP in the Appalachian region of the Eastern United States (20) was used to discover that gain-of-function mutations in the cationic trypsinogen gene (*PRSS1*) could cause HP (21, 22). This discovery implicated genetics as a cause of CP, but also provided a framework for the study of complex, acquired inflammatory disorders. Three of the key observations including: (a) the mutation carriers were 100% normal until they developed an attack of AP, (b) AP sensitized the pancreas in some way so that in most cases RAP followed, and (c) RAP is a driver of CP (23).

### SAPE hypothesis

Based on this model we developed the “Sentinel Acute Pancreatitis Event” hypothesis model (24). The hypothesis is that an episode of AP alters the pancreas to make it hypersensitive to RAP. The mechanisms were hypothesized to represents the resolution to an intense inflammatory event that includes residual pro-inflammatory macrophages (e.g. Th1 → F0E0Th2) and/or other immune cells throughout the parenchyma, and possibility epigenetic changes to the acinar or duct cells that are potentially pro-inflammatory. Thus, a minor increase in stress or injury that was originally only sufficient to generate sub-clinical stress signals that were compensated for by normal pancreas defense mechanisms, now triggers RAP and drives CP after the sentinel acute pancreatitis event. If the stress or injury is minimized, then the new lower inflammatory trigger threshold is not exceeded, the non-necrotic pancreas recovers and functions normally.

## Evidence supporting the SAPE hypothesis

### Alcohol and rats

Our laboratory tested the SAPE hypothesis in an alcohol-consuming rat model. Rats that consumed large amounts of alcohol for months had no pathologic evidence of pancreatitis, even though they did show evidence of mitochondrial stress (25) and neurohormonal compensation for the effects of alcohol (26, 27). Alcohol-consuming rats and rats on similar chow without alcohol were given an episode of AP using cerulean injections (19). After the first episode of AP the histology of the pancreas was similar in alcohol vs. control diet rats. However, after inducing 3 episodes of AP, the alcohol-consuming rats developed severe pancreatic injury, marked infiltration of leukocytes and fibrosis characteristic of CP, whereas the control rats recovered and had minimal pathology. This demonstrated the importance of AP in initiating the process leading to CP that is driven by continued alcohol and RAP, and the fact that alcohol was contributing to this process by altering the type or severity of the immune response in some ways.

### GEMMs and RAP

A mechanism for the SAPE hypothesis was recently demonstrated in mice. Using genetically engineered mouse models (GEMMs) Geisz and Sahin-Toth (28) identified *persistently infiltrating macrophages* after the initial acute pancreatitis event and provided supporting evidence that residual inflammatory cells contribute to the mechanism of enhanced injury and more severe inflammatory response during successive episodes of AP or stressors. Thus, after an initial episode of AP, the pancreas is “primed” for RAP by macrophages.

### Alcoholic pancreatitis in Japan

Takeyama published an important observational cohort study in Japanese after an episode of AP with a 13–17 year follow-up (29). He observed a high rate of RAP and progression to CP in patients following AP who continued alcohol use, with a reduction in RAP and CP in patients who stopped or significantly reduced drinking. This demonstrates that alcohol drives RAP and CP at high rates in patient *after* an initial episode of AP.

### Biliary RAP

The most common cause of AP is impacted gallstones at the sphincter of Oddi beyond the convergence of the common bile duct and the main pancreatic duct. The risk of RAP is very high in these patients, and cholecystectomy (CCY) is recommended as soon as possible, even *during* the sentinel AP admission. Comparing patients with and without CCY suggest (a) the rate of RAP is ~30%, that (b) is reduce to ~11% with CCY (30). However, the rate of AP in controls (e.g., the general population) with gallbladders *in situ* was not calculated (i.e., the risk of AP is *only* at a very high level *after* the sentinel acute pancreatitis event).

### Hypertriglyceridemic RAP

Hypertriglyceridemia (HTG) is associated with AP (31, 32) and CP (33). The upper limits of normal for serum triglyceride (TG) is 150 mg/dL. The risk of HTG-AP is proportional to the serum lipids (34), yet <10% of patients with persistent TG>2,000 mg/dL for years ever have an episode of AP. In contrast, at least a third of patients with HTG that had one episode of AP rapidly develop RAP (34), indicating hypersensitivity of AP patients to RAP.

### PS-cystic fibrosis RAP

An observational study from France in adults with pancreas sufficient cystic fibrosis (PS-CF) found that out of 40 patients followed over their lifetime, 19 (47.5%) had at least one episode of AP, and of the 19 with AP, 15 had RAP (78.9%) (35). This suggest that in adult PS-CF patients, AP occurs in a large minority, but if that have one episode of AP, they are likely to have RAP.

### Timing of interventions to prevent RAP

Four examples of common etiologies of AP and RAP were noted above: Alcohol, biliary, HTG and RAP in PS-CF. Note that strong clinical intervention is implemented AFTER the first attach of AP, as AP sensitizes the pancreas to RAP with low-level injury and stress exposure. For example, alcohol drinking and smoking are not strongly discouraged for prevention of AP, but they are strongly discouraged *after* the first attack of alcoholic AP, as patients are now at very high risk of alcoholic RAP and CP. The same is true for gallstone pancreatitis. While this is the #1 risk factor of AP, surgeons do not take the gallbladder out of all patients in the population to *prevent AP*. Instead, they take the gallbladder out *after* the first episode of AP, recognizing that the patient is NOW at very high risk of RAP. We argue that it is not justified to put patients with high-risk *CFTR* genotypes (including PS-CF) on CFTR-modulators to prevent the first attack of AP. Instead, patients with high-risk *CFTR* genotypes may benefit from CFTR-modulators *after* the sentinel AP attack because they are now at high risk of RAP and CP (36).

## Link between RAP and CP

As noted above, the model of RAP to CP is evident in families with hereditary pancreatitis. Yadav et al. conducted a population-based study in Pittsburgh to determine the outcome of patients after their sentinel pancreatitis event (37). They demonstrated that after AP, RAP occurred most common among alcoholics, intermittent with genetic and idiopathic etiologies, and least among gallstone pancreatitis, with the recurrence directly proportional to duration between AP and CCY. These data were replicated by the Dutch Pancreatitis Study Group, with additional risk shown for smoking (38). This RAP → F0E0CP progression was noted in multiple etiologies, consistent with the idea that the pancreas is sensitized to the AP event, not the inciting stressor or type of injury. Thus, the SAPE phenomenon appears to be similar, regardless of etiology.

## Mechanistic definition of CP and progressive model of symptom development

The SAPE Model was developed to test the hypothesis the CP requires triggering an acute inflammatory event that resolves, but results in hypersensitivity to RAP and recruits/activates resident tissue immune cells that drive continued inflammation, fibrosis, atrophy and other features of CP. The next problem is to more accurately define the CP syndrome in a mechanistic way so that it could be used for early diagnosis and for the exclusion of other disorders and diseases within the differential diagnosis that have similar features. An international task force was commissioned by the European Pancreas Club to develop a consensus definition of CP, and the following mechanistic definition, in two parts (essence and characteristics), was generated (39).

### Mechanistic definition of CP

- *Essence*: Chronic pancreatitis is a pathologic fibro-inflammatory syndrome of the pancreas in individuals with genetic, environmental and/or other risk factors who develop persistent pathologic responses to parenchymal injury or stress.
- *Characteristics*: Common features of established and advanced CP include pancreatic atrophy, fibrosis, pain syndromes, duct distortion and strictures, calcifications, pancreatic exocrine dysfunction, pancreatic endocrine dysfunction and dysplasia.

This definition recognizes the complex nature of CP, separates risk factors from disease activity markers and disease endpoints, and allows for a rational approach to early diagnosis, classification and prognosis (39).

**Figure 1:**
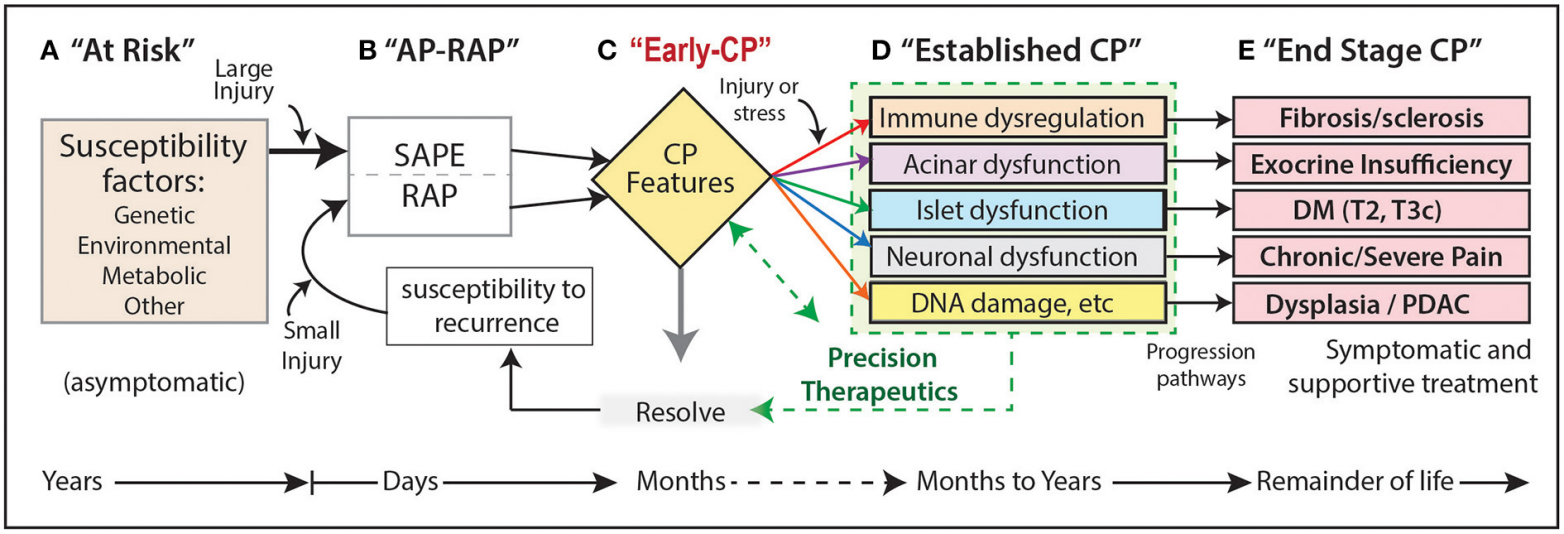
**(A–E)** Progressive CP pathogenesis. Five progressive stages can be defined that persist for days to many years. Each stage may have unique risk factors or stress/injury mechanisms, as well as innate compensatory or protective mechanisms that may be altered or defective in patients who progress. Stage B is a critical driver of the CP pathways as the initial episode of AP (SAPE) lowers the threshold for RAP. CP, chronic pancreatitis; DM, diabetes mellitus; RAP, recurrent acute pancreatitis; SAPE, sentinel acute pancreatitis event; PDAC, pancreatic ductal adenocarcinoma; T2, type II DM; T3c, type IIIc diabetes caused by exocrine pancreas pathology or surgery.

Next, a progressive CP pathogenesis model was proposed to organize the risk, activities and stage of the CP process (Figure 1) (39).

This model illustrates the concept that CP is the result of progression from no disease (A. At Risk) to pancreatic parenchymal destruction (E). Cases typically start with acute pancreatitis (AP, “sentinel AP event” SAPE) and recurrent AP (RAP) stage B. Damage to acinar and duct cells (C-D), and fibrosis (immune cells), leads to diabetes (islet cells), pain syndromes (nervous system) and dysplasia / pancreatic ductal adenocarcinoma (PDAC). The opportunity to intervene is between stages B and D.

### Biomarkers

One of the remaining challenges is to define the biomarkers of disease stage. The first observation is that the progressive model has multiple stages, with unclear transitions between Early CP, Established CP or End-Stage CP. Distinction between stages requires the use of biomarkers to serve as objectively measured characteristics of the underling biological processes. Biomarkers can be clinical features, biochemical analytes, measures of physiologic features or functions, histologic features, imaging studies or other. As multiple cell types are involved in chronic pancreatitis (acinar, duct, islet, immune, nervous, etc.,), biomarkers of each component are needed. Furthermore, criteria on distinctions between stages are yet to be defined.

## Risks leading to CP and opportunities for interventions

The mechanistic definition of CP indicates that CP only occurs in patients with “genetic, environmental and/or other risk factors”. Multiple “other factors” were noted above (e.g. alcohol, gallstones, genetics [*PRSS1, CFTR* variants], and HTG). Based on the evidence surrounding alcohol-associated CP, it appears that a combination of factors may be required to develop AP, RAP and then CP, with progression occurring only in patients “at risk” at sequential stages.

To understand the risks and mechanisms of disease in individual patients Whitcomb and Etemad developed an organized list of etiologies based on Toxic-metabolic factors (including alcohol, smoking, HTG, hypercalcemia), Idiopathic, Genetic, Autoimmune, Recurrent acute and severe acute pancreatitis, and Obstruction (TIGAR-O) (40), which was updated in 2019 (41). This system was designed to be used within the case report forms (CRF) of the *North American Pancreatitis Study 2* (NAPS2, ClinicalTrials.gov NCT01545167), a prospective observational cohort study of over 1,500 RAP/CP patients and 1,250 controls (33, 42, 43). This study provided many new insights, including demonstrating that less than half of CP subjects are very heavy alcohol users (15, 33, 44), and that there were the high rates of *CFTR* variants in RAP and CP patients (45, 46). The presence of risk factors for RAP/CP also differs dramatically between patients in the UK Biobank (47). These example highlight the importance of comprehensive evaluation of patients and observing the primary and secondary combinations of risk factors that generate pathogenic conditions.

*In summary*, the key to understanding the risk of RAP and CP is the radical changes to the pancreas after in initial attack of AP (the SAPE). Although the pancreas is hypersensitized to injury or stress signals after SAPE, reducing or elimination the major injury- or stress-inducing factors markedly diminishes the rate of RAP and, by extension, CP. Thus, etiology- and/or pathway-based, targeted interventions are needed for personalized care of patients with inflammatory diseases of the pancreas.

## Studies CFTR modulators in RAP

Cystic fibrosis (CF) is a well-defined genetic disorder caused by biallelic mutations in the *CFTR* gene with specific diagnostic criteria (48). Some CF patients have a milder course and rather than suffering a complete destruction of the pancreas *in utero* and infancy to become exocrine pancreatic insufficient (PI), they maintain enough pancreatic function to digest food, i.e., exocrine pancreatic sufficient (PS). The high rate of AP and RAP in PS-CF patients was noted above (35). Studies of CF families and wide availability of *CFTR* genetic testing in various populations reveal that *CFTR* variants are also associated with a wide variety of disorders that do not meet criteria for CF, especially CP (including RAP), bronchiectasis and male infertility (49–51). These single-organ disorders are called CFTR-related diseases (CFTR-RD).

### Case series on the use of CFTR modulators in CF patients with RAP

An exciting observation is that patients with PS-CF and RAP often have marked reduction RAP episodes when they are on ivacaftor, a CFTR-potentiator (36, 52–54). These case reports and case series provide compelling evidence that CFTR-modulators (like ivacaftor) may be useful in patients with RAP and early CP when the etiology includes damaging *CFTR* genetic variants (55).

### Population-based studies of CFTR modulators in CF patients

The problem of population-based studies is that the “case-control” design limits insights into the true, complex pathophysiologic mechanism of pancreatic disease. For example, 2021 Ramsey, et al. published, “Cystic Fibrosis Transmembrane Conductance Regulator Modulator Use Is Associated With Reduced Pancreatitis Hospitalizations in Patients With Cystic Fibrosis” (56). This study used an administrative database, *MarketScan*, from 2012 to 2018 to evaluate AP hospitalizations and CFTR modulator use among patients with CF. In summary, they found 10,417 patients with CF, including 1,795 who received a CFTR modulator, and classified patients as PS-CF or PI-CF based on pancreatic enzyme replacement therapy (PERT) use. AP was more common in PS-CF than PI-CF (2.9 vs. 0.9%, P = 0.007), and use of CFTR-modulators significantly reduced the frequency of AP events by 67%. However, they estimated that CFTR-modulator use would only reduce AP in PS-CF patients from *10.20 to 3.26 per 1,000 patient-years*. Thus, the justification to use CFTR-modulators in CF patients for the prevention of the initial attack of AP is minimal. However, they did not consider the role of the SAPE model, problems in patient classification using administrative codes, capturing pre-existing AP, a short time frame to capture RAP in this cohort (see below).

There are always limitations to administrative database studies. In this case AP was reported in several patients with pancreatic insufficient CF (PI-CF), highlighting potential classification problems. The incidence of AP in PI-CP should be negligible since these patients have nearly complete loss of trypsin-secreting acinar cells. They used PERT as a surrogate of PI, but PERT is used both for exocrine pancreatic insufficiency (EPI) and pain, bloating, diarrhea and other reasons in pancreatitis patient—especially young ones (57). Thus, many of the PI-CF patients were likely PS-CF with misclassification, reducing the power of the relevant study (i.e., AP in PS-CF).

The etiology of AP in these patients is also unknown. Patients with CF are unlikely to have alcohol-related pancreatitis but have an increased risk of biliary disease and gallstones. Thus, the incidence of AP in the patients using a CFTR-modulator may be artificially increased by gallstone AP, therefore decreasing the estimate of the true effect CFTR-modulators on decreasing AP events.

Another limitation of the database study is that it did not allow the investigators to determine who had AP *before* the 4–6 year observational time frame (e.g. who was already a RAP patient). The study was designed to examine the effect on reducing *the initial attack of AP* in a population with a slightly higher risk of AP than expected in the general population (58, 59), but does not address the primary problem of the very high risk of RAP in patients who had AP. Based on the point above, the study could be framed as a reduction of AP events in 5 patients with RAP.

Finally, the administrative databases included AP events during a short time window. The incidence of AP in the CF population during the study period (~4 years of observation per patient, page 2449–2450) was [22 + 145]/10,417 = 1.6%. The incidence of RAP in RAP patients is 100% (by definition). Of the 8 PS-CF subjects with AP, there were 5 additional attacks (RAP) noted within a 3.9-year observation window. If no individual had more than 2 attacks, then 62.5% were RAP patients—even though the PS-CF patients were on CFTR-modulators 36.5% of the time. Furthermore, not all patients appeared in the database at the same time, and follow-up was limited and variable (median follow-up was 3.9 +/– 2.1 years).

In summary, careful consideration is needed in evaluating the role of medications on RAP and CP based on study design and patient classification. The very strong effect of CFTR modulators reducing RAP rate is shown in case series studies with nearly every patient responding. However, in a retrospective administrative database study, the question that was asked (do CFTR modulators reduce the rate of initial attack of pancreatitis in patients al low individual risk) may not answer more specific patient questions needed to address a precision medicine approach. The SAPE model demonstrates that patients with a previous AP attack are highly susceptible to RAP. And in contrast to expected rates of AP in the CF population (10.2 per 1,000 patient years), *RAP patients develop AP at rates between 250 and 1,000 per 1,000 patient years*, with some patients having 3–4 attacks per year. These are the pancreatitis patients that precision medicine is designed to help.

## Other etiologies of CP

CFTR-related pancreatitis represents an illustrative approach to etiology-based disease management using highly targeted therapy. The SAPE provides a clear trigger for immediate evaluation and prevention of RAP. Other areas of need are preventative or therapeutic treatments for CP caused by *PRSS1* gain-of-function mutations, other trypsin-related disorders (e.g., simple and complex genetic variants linked to serine protease inhibitor Kazal-type 1, *SPINK1*, or *Chymotrypsin-C, CTRC*), ER stress-related CP, hypertriglyceridemia recurrent acute pancreatitis and others. It is unclear whether it will be possible to repurpose existing drugs or develop gene-based therapies in the future, but the framework presented here for CFTR-RD is a clear direction toward success.

## Conclusions

A transition is required from the old CP disease paradigm to a new paradigm for evidence-based guidance on early identification of patients at risk of RAP and CP. A detailed understanding of the disease risks and stage are needed to determine which mechanism and pathways are pathogenic, and to choose appropriate therapies to prevent RAP and CP. The new Mechanistic Definition of CP, the Progressive Pathogenesis Model and the SAPE phenomenon are critical for designing new intervention studies. These new insights indicate that (a) AP transforms a patient into a high-risk group for RAP and CP, (b) interventions are justified in patients with a history of AP to prevent RAP, (c) evidence from a case series on PS-CF patients with RAP and CFTR-modulators justifies new, scientifically rigorous clinical intervention trials with CFTR-modulators and (d) selecting patients with previous AP or RAP and damaging CFTR variants allows for a well powered study with a limited number of patients based on the high effect size of CFTR-modulators and the high rate of AP events in patients with a history of AP or RAP.

## Author contributions

The author confirms being the sole contributor of this work and has approved it for publication.

## Funding

This work was primarily funded through the National Institutes of Diabetes and Digestive and Kidney Diseases (Grant No. DK061451 to DW).

## Conflict of interest

Author DW is a consultant to Nestle', Regeneron and Ariel Precision Medicine. He is a co-founder of Ariel Precision Medicine and may have equity.

## Publisher's note

All claims expressed in this article are solely those of the authors and do not necessarily represent those of their affiliated organizations, or those of the publisher, the editors and the reviewers. Any product that may be evaluated in this article, or claim that may be made by its manufacturer, is not guaranteed or endorsed by the publisher.
